# A Randomized Control Trial Comparing 2 Levofloxacin-Containing Second-Line Therapies for *Helicobacter pylori* Eradication

**DOI:** 10.1097/MD.0000000000003586

**Published:** 2016-05-13

**Authors:** Seng-Kee Chuah, Chih-Ming Liang, Chen-Hsiang Lee, Shue-Shian Chiou, Yi-Chun Chiu, Ming-Luen Hu, Keng-Liang Wu, Lung-Sheng Lu, Yeh-Pin Chou, Kuo-Chin Chang, Chung-Huang Kuo, Chung-Mou Kuo, Tsung-Hui Hu, Wei-Chen Tai

**Affiliations:** From the Division of Hepatogastroenterology, Department of Internal Medicine, Kaohsiung Chang Gung Memorial Hospital (S-KC, C-ML, S-SC, Y-CC, M- LH, K-LW, L-SL, Y-PC, K-CC, C-HK, C-MK, T-HH, W-CT); Chang Gung University, College of Medicine, Kaohsiung, Taiwan. (S-KC, C-HL, Y-CC, K-LW, K-CC, T-HH, W- CT); Division of Infectious Diseases, Department of Internal Medicine, Kaohsiung Chang Gung Memorial Hospital (C-HL).

## Abstract

Summary of Trial Design.

Lengthy exposure to quinolone-containing triple therapy in *Helicobacter pylori* eradication leads to the development of drug resistance. Sequential therapy with a quinolone and metronidazole -containing regimen appears to be an effective treatment option. This randomized controlled trial aimed to compare the efficacy of 5-plus 5 days’ levofloxacin and metronidazole-containing sequential therapy (EALM) with that of 10-day levofloxacin-containing triple therapy (EAL) in second-line *H pylori* eradication treatment.

One hundred and sixty-four patients who had failed the *H pylori* eradication attempts using the standard triple therapy (proton pump inhibitor bid, clarithromycin 500 mg bid, amoxicillin 1 g bid × 7 days) were randomly assigned to either an EALM therapy group (n = 82; esomeprazole 40 mg bid and amoxicillin 1 g bid for 5 days, followed by esomeprazole 40 mg bid, levofloxacin 500 mg qd, and metronidazole 500 mg tid, for 5 days) or a 10-day EAL therapy group (n = 82; levofloxacin 500 mg qd, amoxicillin 1 g bid, and esomeprazole 40 mg bid). One patient was lost to follow-up in each group. Follow-up for *H pylori* status was performed 4 to 8 weeks later.

Eradication rates for the EALM and EAL groups were 90.2% (74/82, 95% confidence interval [CI] = 83.7%–96.8%) and 80.5% (66/82, 95% CI = 71.7%–89.2%, *P* = 0.077) in the intention-to-treat analysis; and 91.4% (74/81, 95% CI = 85.1%–97.6%) and 81.5% (66/81, 95% CI = 72.8%–90.1%, *P* = 0.067) in the per-protocol analysis. The adverse events for the EALM and EAL groups were 23.5% versus 11.1%, *P* = 0.038 but were all very mild and were well tolerated except for 1 patient with poor compliance. The compliances were 98.8% and 100%, respectively, between the 2 groups. An antibiotic resistance to levofloxacin was the clinical factor influencing the efficacy of *H. pylori* eradication therapy in the EAL group, and dual resistance to levofloxacin and metronidazole in the EALM group.

Levofloxacin and metronidazole-containing sequential therapy achieved a >90% eradication rate as a second-line *H pylori* therapy. Dual antibiotic resistance to levofloxacin and metronidazole was the clinical factor influencing the efficacy of *H pylori* eradication therapy in the sequential therapy (ClinicalTrials.gov number: NCT02596620).

## Abstract 2

Supplemental Digital Content is available in the text

## INTRODUCTION

Quadruple therapy—consisting of a proton pump inhibitor (PPI), a bismuth salt, metronidazole and tetracycline—are recommended by the Maastricht IV/Florence-Consensus Report (2011/2012) and the second Asian Pacific Consensus Report.^1,2^ However, the failure rates of quadruple therapy in Taiwan have reached as high as 20%.^3,4^ Furthermore, bismuth salts are not available in many hospitals. International consensus guidelines^1,2,5^ on the management of *Helicobacter pylori* infection have recommended a levofloxacin-containing second-line triple therapy consisting of a PPI, a quinolone, and amoxicillin after previous eradication failures because this particular therapy exhibits remarkable in-vitro activity against *H pylori*.^6,7^ In addition, an in-vivo synergistic effect is exhibited with respect to quinolone antimicrobial agents and PPIs when strains of *H pylori* are targeted.^7–9^ Moreover, quinolone is an effective orally administered antimicrobial drug and is well tolerated, which is important because—aside from antibiotic resistance—compliance plays a cardinal role in eradication. Extending the duration of treatment to at least 10 days improves eradication rates.^10,11^ Unfortunately, the lengthy use of quinolone is strongly associated with a risk of developing bacterial resistance.^12^ Besides, lengthy exposure to quinolone could be associated with delays in diagnosing tuberculosis and with the development of drug resistance.^13^

Therefore, the search for an ideal second-line rescue therapy is mandatory. Sequential therapy has been widely used to treat first-line *H pylori* infection. However, few reports exist on sequential therapy comprising only 5 days of levofloxacin as a second-line *H pylori* eradication regimen, and none had compared a new regimen with the conventional quinolone-containing triple therapy. However, ofloxacin combined with metronidazole shows a synergistic effect against bacteria, and high-dose metronidazole can overcome metronidazole resistancy.^6,14^ We hypothesized that the addition of metronidazole may enhance the effectiveness of levofloxacin-containing therapy, and that the shortening of levofloxacin treatment to 5 days could consequently minimize the impact on tuberculosis. Therefore, we conducted this randomized, controlled trial to compare the efficacy of 5-plus 5 days’ levofloxacin- and metronidazole-containing sequential therapy (EALM) with 10-day levofloxacin-containing triple therapy (EAL) in second-line *H pylori* eradication treatments, and to determine the influencing clinical factors.

## METHODS

### Ethics Statement

This prospective randomized trial was conducted in Kaohsiung Chang Gung Memorial Hospital (outpatient department) in Southern Taiwan. This protocol was approved by the institutional review board and the Ethics Committee of Chang Gung Memorial Hospital (IRB103–2581C). All patients provided their written informed consent before enrollment. None of our patients belonged to the minors’/children's group. The ClinicalTrials.gov registration identifier is NCT02596620.

### Trial Design and Settings

#### Participants

We invited 210eligible *H pylori*–infected outpatients at least 18 years’ old with endoscopically proven peptic ulcer diseases or gastritis who had failed first-line eradication therapies with standard triple regimens (PPI twice daily, 500 mg of clarithromycin twice daily, and 1 g of amoxicillin twice daily) to join the study (supplementary material). When at least 2 positive results were noted on rapid urease test, histology, and ^13^C-urea breath test, or a positive culture result occurred following first-line treatment, eradication failure was confirmed.^4^ We enrolled 164 patients with treatment failure after excluding those who had taken antibiotics, bismuth, PPIs, or nonsteroidal anti-inflammatory drugs within the previous 4 weeks, were allergic to the medications used, had a history of previous gastric surgery or serious concomitant illness, or were currently pregnant.^15^

### Selection of Patients

**FIGURE 1:**
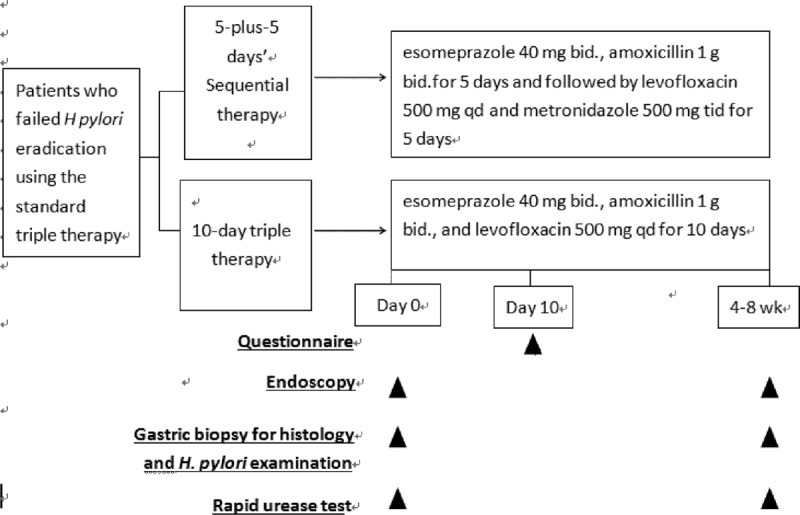
Schematic flowchart of study design.

Figure 1 showed the schematic flowchart of study design. The *H pylor*i–infected outpatients who had failed the first-line eradication therapies were randomly assigned to undergo 5-plus-5 days’ EALM regimens (n = 82; esomeprazole 40 mg bid and amoxicillin 1 g bid for 5 days, followed by esomeprazole 40 mg bid, levofloxacin 500 mg qd, and metronidazole 500 mg tid for 5 days) or 10-day EAL regimens (n = 82; levofloxacin 500 mg qd, amoxicillin 1 g bid, and esomeprazole 40 mg bid). Drug compliance and adverse events were checked on day 11. We gave patients with peptic ulcers an additional 3 weeks of esomeprazole 40 mg orally once daily.^15^ The second endoscopy to assess the effect of *H pylori* eradication treatment was performed 4 to 8 weeks later, along with a rapid urease test and histological examination. An alternate urea breath test was performed when a patient was reluctant to undergo another endoscopy.^16^ The technicians who performed the *H pylori* tests (rapid urease test and urea breath test) or who filled in the questionnaires, as well as the pathologists, were blinded to the eradication regimens that the patients received. Successful second-line eradication was achieved when the patient was found to have a negative result for both the rapid urease test and histology or a negative urea breath test.^16^

A complete medical history and demographic data were obtained from each patient, including age, sex, medical history, smoking history, alcohol intake, and tea or coffee consumption. Smoking was defined as consumption of ≥1 packs of cigarettes per week. Adverse events that interfered with the patients’ daily life quality were prospectively evaluated, such as abdominal pain, diarrhea, constipation, dizziness, taste perversion, headache, anorexia, nausea, vomiting, and rash. Compliance was checked by counting unused medication at the completion of treatment. Poor compliance was defined as taking <80% of the total medication.^17,18^

### Objectives

The present study aimed to determine the efficacy of 5-plus 5 days’ EALM therapy for patients after failure of standard triple therapy, and its influencing clinical factors, by comparing it with 10-day EAL therapy as second-line therapy.

### Outcomes

The primary endpoint of our study was the successful eradication of *H pylori*. We also conducted additional analyses on adverse events during the therapies.

### Culture and Antimicrobial Resistance

*H pylori* isolation biopsy specimens were obtained from the gastric antrum and corpus for culture in a medium containing brucella chocolate agar with 7% sheep blood, and incubated for 4 to 5 days under microaerobic conditions.^19^ The minimal inhibitory concentration (MIC) was determined by the agar dilution test. *H pylori* strains with MIC values ≥0.5, ≥1, ≥1, ≥4, and ≥8 mg/L were considered to be the resistant breakpoints for amoxicillin, clarithromycin, levofloxacin, tetracycline, and metronidazole, respectively.

### Randomization

We used a computer-generated randomization list to get a “random sequence” and combine blocking and stratified randomization strategies.^4^ We used separate randomization procedures within each of 2 participant groups a ratio of 1:1. We then set a block for every 6 participants. The statistician generated a randomization list, and the doctors determined the patients who were suitable for this study and sorted sequence order, which was provided in a blinded manner by a research assistant. Each patient received the medications on the same day from the pharmacy of our hospital. The researchers revealed the code only after the entire study had been completed.

### Statistical Methods

According to previous publications,^4,19^ the eradication rate for the EAL group was 70%, and we assumed that the EALM group could achieve 90% eradication rate. That would imply a 20% increase in eradication rates. To attain a statistical power of 90%, the present study should include at least 80 subjects in each group. Therefore, if 95% of patients could complete the follow-up, we would be able to achieve the 2-sided *P* value of 0.05.

All the statistical analyses were performed using the SPSS program (Statistical Package for the Social Sciences version 18, SPSS Inc, Chicago, IL). We chose the eradication rate, presence of adverse events, and level of patient compliance as the primary outcome variables.^19^ In the present study, *χ*^2^ tests with or without Yates’ correction for continuity and Fisher exact tests were used to analyze the outcomes. Intention-to-treat (ITT) and per-protocol (PP) approaches were applied in the analysis. Patients in the ITT analysis included all patients who started any dose after entry into the study. We considered patients with unknown infection status or who were lost to follow-up in the ITT group as treatment failures. However, we excluded patients with unknown *H pylori* status following therapy and those with major protocol violations in the PP analysis.^19^ Statistical significance was considered to be a *P* value <0.05. We also performed univariate and multivariate analyses on the clinical and bacterial parameters for each group of patients to determine the clinical factors for treatment outcome.

## RESULTS

**FIGURE 2:**
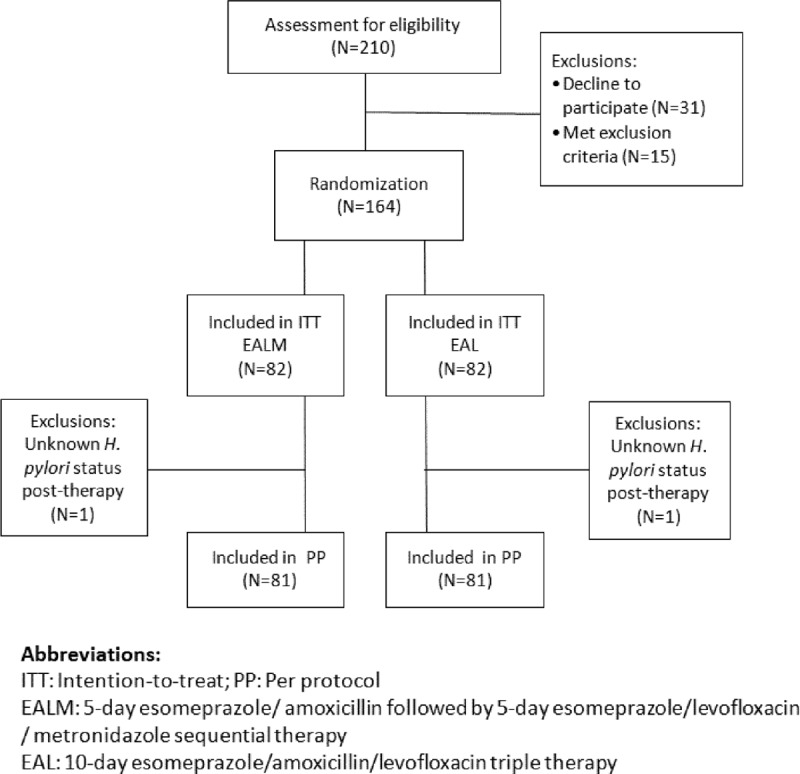
Patients’ deposition.

From October 1, 2013 to September 30, 2015, a total of 210 eligible patients with endoscopically proven peptic ulcer diseases or gastritis who failed first-line eradication therapies with standard triple regimens were invited to take part in the study, of which 164 patients were enrolled (n = 82 per group) in the ITT analysis. Ultimately, 1 patient was lost during follow-up in each group, resulting in 81 patients in the PP study for the EALM group and 81 for the EAL group (Figure 2). The demographic data of the 2 groups are summarized in Table 1; none of the variables was significantly different between the groups.

**TABLE 1:**
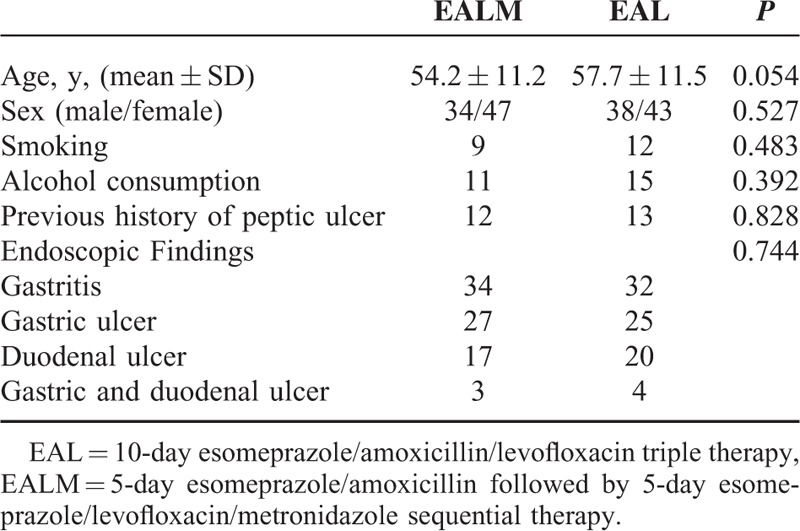
Demographic Data and Endoscopic Appearances of the 2 Patient Groups

The eradication rates for the EALM and EAL groups were 90.2% (74/82, 95% confidence interval [CI] = 83.7%–96.8% and 80.5% (66/82, 95% CI = 71.7%–89.2%, *P* = 0.077) in ITT analysis, and 91.4% (74/81, 95% CI = 85.1%–97.6%) and 81.5% (66/81, 95% CI = 72.8%–90.1%, *P* = 0.067) in PP analysis, respectively (Table 2).

**TABLE 2:**
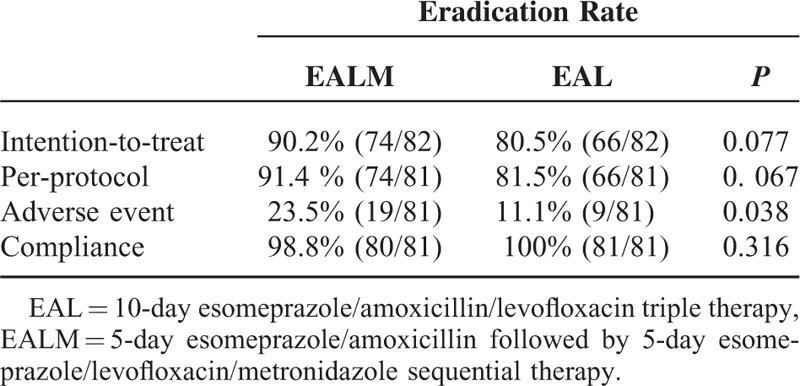
Major Outcomes of Eradication Therapy

### Adverse Events and Compliances

The adverse events are summarized in Table 3. More patients experienced adverse events in the EALM group (23.5%) than in the EAL group (11.1%; *P* = 0.038), as shown in Table 2. The drug compliances of both groups were excellent (98.8% in the EALM group vs 100% in the EAL group).One patient failed to finish the medications because of severe headache and nausea after taking metronidazole

**TABLE 3:**
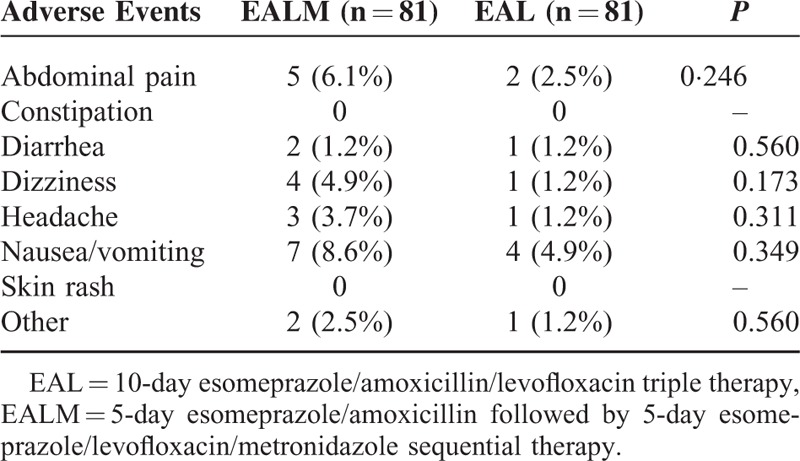
Adverse Events During Eradication Therapies

### Antibiotic Resistance

**FIGURE 3:**
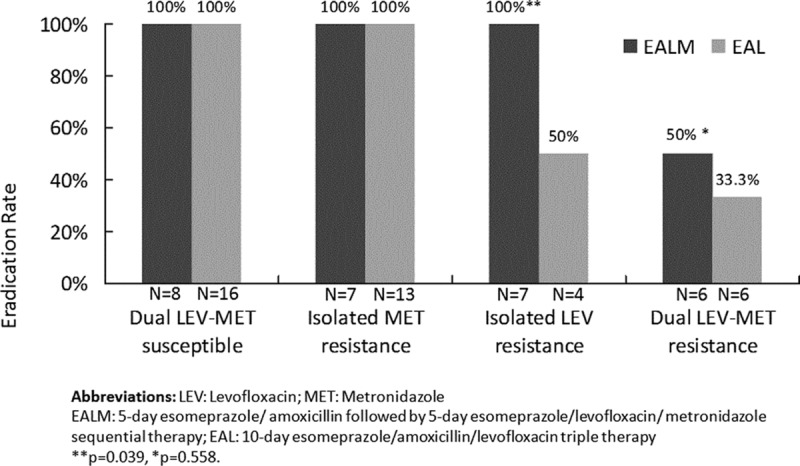
The effects of levofloxacin and metronidazole resistance on *Helicobacter pylori* eradication rates.

The positive *H pylori* culture rate was 71.2% (68/94) and the antibiotic resistance rate for levofloxacin was 33.8% (23/68) in the present study. Overall, the *H pylori* eradication rates for the levofloxacin-susceptible strains and levofloxacin-resistant strains were 100% (44/44) and 60.9% (14/23) in the PP analysis. However, the antibiotic resistance rate for metronidazole was 48.5% (33/68). The *H pylori* eradication rates for the metronidazole-susceptible strains and metronidazole-resistant strains were 94.3% (33/35) and 75.8% (25/33), respectively. Among the 33 patients with metronidazole-resistant strains, 1 patient had concomitant resistance to amoxicillin and he failed the eradication. If we analyzed these data separately according to the EALM and EAL groups, all patients with strains susceptible to both levofloxacin and metronidazole (dual LEV-MET susceptible) and with isolated metronidazole resistance were eradicated of their strains (Figure 3). Also, all patients with isolated levofloxacin resistance showed eradication in the EALM group, compared with only 50% in the EAL group (*P* = 0.039). The eradication rates for patients with strains resistant to both levofloxacin and metronidazole (dual LEV-MET resistance) were 50% in the EALM group compared with 33.3% in the EAL group (*P* = 0.588). However, as the amoxicillin resistance rate was only 0.01% (1/68) in the present study, we did not include it in the sub-analysis.

### Factors Influencing the Efficacy of *H pylori* Eradication

For the EALM group, the univariate analysis showed that drug compliance (*P* < 0.001), amoxicillin resistance (*P* = 0.011), metronidazole resistance (*P* = 0.026), and dual resistance to both levofloxacin and metronidazole (*P* = 0.004) were the clinical factors influencing the efficacy of *H pylori* eradication therapy (Table 4). Multivariate analysis revealed that only dual resistance to both levofloxacin and metronidazole (*P* = 0.018) was an independent risk factor for eradication failure in the EALM patients (Table 5). For the EAL group, the univariate analysis showed that metronidazole resistance (*P* < 0.001) and dual resistance to both levofloxacin and metronidazole (*P* < 0.001) were the clinical factors influencing the efficacy of *H pylori* eradication therapy.

**TABLE 4:**
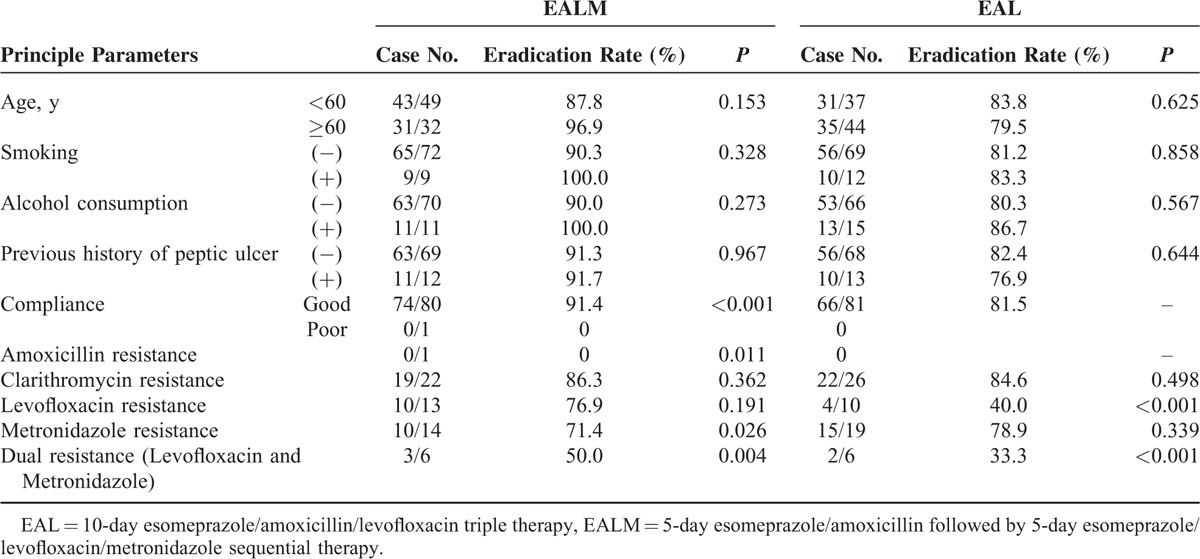
Univariate Analysis of the Clinical Factors Influencing the Efficacy of *Helicobacter pylori* Eradication

**TABLE 5:**
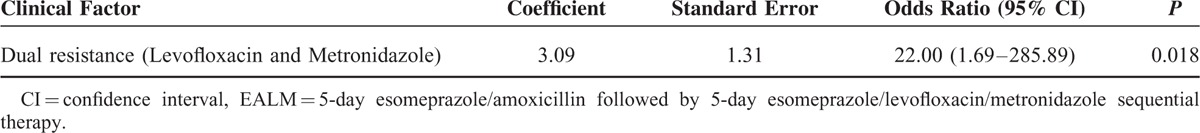
Multivariate Analysis of the Clinical Factors Influencing the Efficacy of *Helicobacter pylori* Eradication in EALM Group

## DISCUSSION

In our study, we observed that EALM therapy could achieve a success rate of >90% in both ITT and PP analysis. This was not achieved with EAL therapy. Importantly, we observed that all patients with isolated metronidazole resistance and isolated levofloxacin resistance were eradicated in the EALM group. There was no obvious difference in compliance between the 2 groups, although more side-effects were observed in the EALM group. The results revealed that 5-plus 5 days’ metronidazole and levofloxacin-containing sequential therapy was effective in areas with high resistance.

Generally, the choice of a second-line treatment depends on which treatment was used initially, as it was concluded that re-treatment with the same regimen should not be recommended.^6,20,21^ In the present study, levofloxacin was not prescribed in the first-line therapy. It should be an ideal second-line regimen as recommended by many consensus reports.^1,2,5^ Duration of EAL therapy was reported to be an important factor for successful eradication with a preference of 10-day regimens as second-line therapies.^22^ However, our previous report showed that 14-day levofloxacin-containing triple therapies could attain eradication rates >90%.^16^

Resistance to quinolone antimicrobial agents is common because of plasmid-mediated horizontally transferable genes encoding quinolone resistance and has been the key factor for eradication failure.^12,19^ Therefore, when we prescribe levofloxacin-containing regimens, we should always strive to achieve grade “A” or “B” eradication report cards. One of the alternative treatments is to apply tailored therapy according to susceptibility to antibiotics.^19^ However, determination of antibiotic sensitivity by pathogen culturing is time-consuming and the successful culture rate of *H pylori* from clinical specimens is not 100% (rather, 70%–80%).^23^ The use of scientific technology allows the identification of genotypic-resistant point mutations (N87 and D91) in the quinolone resistance-determining region of the *gyrA* gene from gastric biopsy specimens—with a >93% success rate.^24^ Again, genotypic resistance-guided therapy is not yet widely available commercially and may be expensive.

It is important to highlight that, in this study, levofloxacin was used only for 5 days in the 5-plus-5 days’ EALM therapy group and achieved >90% eradication rates. Our results are encouraging because it shortened the levofloxacin exposure to 5 days instead of 10 to 14 days. This study was one of the not numerous studies performed outside Italy, which have shown that sequential treatment is at least 90% efficacious. Sequential therapy has been proposed after the observation that spontaneous mutation rates for resistance to metronidazole and clarithromycin are quite high in many countries.^9^ It is therefore predictable that resistant organisms may be harbored in all patients’ stomachs, which prompted the origin of sequential therapy. The mechanism to explain how sequential therapy works is not well established. Its rationale that the administration of amoxicillin in the first 5 days of treatment is believed to considerably reduces the bacterial density, and thus decreases proportionally the number of chemoresistant bacteria.^25^ The fact that, in the present study, the concomitant treatment with the same drugs in the EAL group is far less efficacious than sequential therapy infers that it is plausible that antibiotics other than amoxicillin could possibly interfere with amoxicillin itself. Hence, selecting the organisms resistant to metronidazole and levofloxacin, which current study showed, was responsible for the therapeutic failure in both treatments.

Years ago, one of the most important recommendations when treating bacterial infectious diseases was to avoid the simultaneous administration of bactericidal and bacteriostatic chemotherapies.^26^ However, practice revealed that to fight *H pylori* infection, both bactericidal and bacteriostatic antibiotics can instead be prescribed at the same time. The coadministration of antibiotics does not depend on whether they are bacteriostatic or bactericidal, but depends on how they act together (antagonistic, synergistic, or additive action).^26,27^ The concomitant use of metronidazole played a crucial role despite the fact that metronidazole resistance was as high as 48.5% in the present study. Our results showed that all patients with isolated metronidazole resistance showed eradication with EALM therapy, but 4 patients with metronidazole resistance failed eradication in the EALM group probably because of concomitant resistance to amoxicillin(n = 1) and levofloxacin (n = 3). Likewise, a 100% eradication rate was achieved with isolated levofloxacin-resistant patients when they were prescribed with EALM therapy, yet only half showed eradication when prescribed with EAL therapy. This may perhaps be explained by the combined ofloxacin and metronidazole prescriptions, which have been reported to have a synergistic effect against anaerobic bacteria.^15^ However, the influence of metronidazole resistance on the eradication rate could be overcome by increasing the dose.^28–30^ This result might be related to the changes in oxygen pressure in the gastric environment, which causes metronidazole-resistant *H pylori* isolates to become metronidazole-susceptible under low oxygen conditions in vitro.^28,31^

Antimicrobial resistance is not the sole explanation for failing eradication. Nonadherence with treatment is a well-recognized factor. A careful evaluation of side effects is mandatory for the success of eradication because side effects influence adherence with treatment regimens. Nevertheless, a 98.8% drug compliance was attained in present study despite the fact that 23.5% of patients in the EALM group had experienced adverse events. The possible explanation could be that the side effects were usually very mild and were well tolerated. Only 1 patient with poor compliance—owing to a severe adverse event after starting the metronidazole—failed the attempt to eradicate *H pylori.* The second reason could be the strategies we undertook to improve drug compliance in patients, which included a comprehensive explanation of the possible side effects of their medications and of the importance of adhering to their eradication therapy. In our experience, compliance with prescribed medications was increased by providing the patients with a consultation phone number.

Another explanation for the high eradication rates in the present study was the use of high-dose esomeprazole (40 mg twice daily) rather than standard-dose therapy. High-dose therapy is less affected by the CYP2C19 polymorphism and thus is better able to increase the intragastric pH and maximize the effects of antibiotics.^32^

This study has several limitations. This is a single-medical-center study and the small populations with antibiotic resistance data included in this study have impeded the evaluation of the effects of antibiotic resistance on eradication efficacy. Large-sample-sized, prospective randomized studies are mandatory to precisely interpret the association between antibiotic resistance and the efficacy of this therapy in Taiwan. Nevertheless, the present study observed that this second-line sequential therapy achieved a >90% eradication rate. Importantly, it solves the important issue of concern, the shortening of the levofloxacin exposure to 5 day is of great benefit to public health. Moreover, the success of the sequential therapy can be attributed to the lack of levofloxacin resistance.

## CONCLUSIONS

Levofloxacin- and metronidazole-containing sequential therapy achieved a >90% eradication rate as second-line treatment for eradication of *H pylori*. Dual antibiotic resistance to levofloxacin and metronidazole was identified as the clinical factor influencing the efficacy of *H pylori* eradication treatment in the sequential therapy.

## Supplementary Material

Supplemental Digital Content
