# Supplementary material for: A Randomized Control Trial Comparing 2 Levofloxacin-Containing Second-Line Therapies for Helicobacter pylori Eradication
Source: Medicine (Baltimore). 2016 May 13;95(19):e3586. doi: 10.1097/MD.0000000000003586 (PMC4902499; doi:10.1097/MD.0000000000003586)
Supplement: Supplemental Digital Content [file medi-95-e3586-s001.doc]

**Study Protocol**

**Title:** **A randomized control trial comparing two levofloxacin-containing second-line therapies for *Helicobacter pylori* eradication**

**Abstract:**

**Background:** The global eradication rate of the standard first line triple therapy has fallen below 80% in many countries. Therefore, several alternative first-line regimens have been proposed in order to reach an eradication rate of 90% or greater. Sequential therapy consisting of a PPI plus amoxicillin for 5 days, followed by a PPI plus clarithromycin and metronidazole for another 5 days, has been shown to be more effective than the standard triple therapy in first-line treatment in Italy and also in Taiwan. Another strategy to overcome the declining eradication rate in first-line therapy is to develop an effective second-line rescue regimen. The rationale for this strategy is that the cumulative eradication rate could be as high as 98% if the first-line and second-line eradication rates were 80% and 90%, respectively. Recently, levofloxacin-based triple therapy was shown to be more effective and better tolerated than quadruple therapy in second- and third-line treatment for *H. pylori* infection. However, even large meta-analyses of fluoroquinolone triple therapy have shown that neither 7 days nor 10 days of therapy provides 90% or better treatment success. Reports describing second line *Helicobacter pylori* eradication using sequential therapy is encouraging. It has been reported that the combination of ofloxacin and metronidazole shows a synergistic effect against anaerobic bacteria and high dose metronidaozole can overcome metronidazole resistancy. We hypothesized that the addition of high dose metronidazole might also lead to a higher eradication rate for levofloxacin-based therapy in the treatment of *H. pylori* infection.

**Aims:** to compare the efficacies of 10-day sequential and 10-day levofloxacin-based triple therapy (PPI+ amoxiccilin+ levofloxacin, EAL) in the treatment for patients after failure of standard triple therapy and to determine what clinical and bacterial factors influencing the efficacy of salvage regimens.

**Methods:** Two hundred patients who fail *H pylori* eradication using the standard triple therapy (proton pump inhibitor bid, clarithromycin 500 mg bid., amoxicillin 1g bid.) are randomizely assigned to either 10-day sequential(EALM) (esomeprazole 40 mg bid., amoxicillin 1 g bid.for 5 days and followed by levofloxacin 500 mg qd and metronidazole 500 mg tid for 5 days) or 10-day EAL (esomeprazole 40 mg bid., amoxicillin 1 g bid., and levofloxacin 500 mg qd) Repeated endoscopy with rapid urease test, histological examination and culture is performed at four to eight weeks after the end of anti-*H pylori* therapy. If patients refused follow-up endoscopy, urea breath tests are conducted to assess *H. pylori* status. The rates of eradication, adverse events and compliance will be compared between groups by chi-square test, and the clinical and bacterial factors influencing the efficacy of salvage regimens are assessed by multivariate analysis.

**Expected results:** *H. pylori*-infected patients will be enrolled and randomly assigned to either 10-day sequential (n=100) or 10-day EAL (n = 100) therapies. Intention-to-treat analysis and the per-protocol analysis were speculated to demonstrate the 10-day sequential treatment can achieve a higher eradication rate. In Taiwan, 10-day sequential is expected to achieve 90% eradication rate.

Multivariate analysis for the clinical and bacterial factors influencing the efficacy of

2nd-line eradication therapy should merit further investigations.

**INTRODUCTION**

The global eradication rate of the standard first line triple therapy has fallen below 80% in many countries (1) Therefore, several alternative first-line regimens have been proposed in order to reach an eradication rate of 90% or greater. Sequential therapy consisting of a PPI plus amoxicillin for 5 days, followed by a PPI plus clarithromycin and metronidazole for another 5 days, has been shown to be more effective than the standard triple therapy in first-line treatment in Italy and also in Taiwan (2-4).

Another strategy to overcome the declining eradication rate in first-line therapy is to develop an effective second-line rescue regimen. The rationale for this strategy is that the cumulative eradication rate could be as high as 98% if the first-line and second-line eradication rates were 80% and 90%, respectively. The most widely used second-line therapy for *Helicobacter pylori (H. pylori)* eradication is quadruple therapy, consisting of a proton pump inhibitor (PPI), a bismuth salt, metronidazole and tetracycline as recommended by the Maastricht IV/Florence-Consensus Report (2011/2012) and second Asian Pacific Consensus Report (5, 6). However, the failure rates of quadruple therapy are increasing. Pretreatment antibiotic resistance is the most important factor in nonresponsive patients to initial treatment (7). In Taiwan, the failure rates of quadruple therapy had reached as high as 20% which were partly relevant to the high antibiotic resistance rates to metronidazole, and clarithromycin in *H. pylori* strains (8-10). Besides, bismuth salts are not available worldwide anymore. Therefore, the search for an ideal second line rescue therapies is mandatory especially the non-bismuth containing regimens. Generally, the choice of a second-line treatment depends on which treatment was used initially, as it would appear that retreatment with the same regimen cannot be recommended (11, 12).

Recently, levofloxacin-based triple therapy was shown to be more effective and better tolerated than quadruple therapy in second- and third-line treatment for *H. pylori* infection. However, even large meta-analyses of fluoroquinolone triple therapy have shown that neither 7 days nor 10 days of therapy provides 90% or better treatment success (13). Recently, Sequential therapy has been widely used in treating first line *H. pylori* infection. However, there are very few reports on sequential therapy as second line *H. Pylori* eradication regimen in the literature. It has been reported that the combination of ofloxacin and metronidazole shows a synergistic effect against anaerobic bacteria (14, 15) and high dose metronidaozole can overcome metronidazole resistance (16). We hypothesized that the addition of high dose metronidazole might also lead to a higher eradication rate for levofloxacin-based therapy in the treatment of *H. pylori* infection. The dosage and duration of levofloxacin used in previous Taiwanese report were 500 mg daily for 7 days and attained unacceptable results of less than 80% eradication rates (9, 10, 17, 18) even when the dosage was increased to 750 mg daily in one study (19).

We therefore conducted current randomized, controlled trial to determine the efficacy of 10-days sequential therapy for patients after failure of standard triple therapy and its influencing clinical factors.

**Materials and Methods**

**Patients and Study design**

A total of 220 consecutive *H. pylori-*infected outpatients, at least 18 years of age, with

endoscopically proven peptic ulcer diseases or gastritis who failed first-line

eradication therapies with standard triple regimens (PPI twice daily, 500 mg of

clarithromycin twice daily and 1 g of amoxicillin twice daily) will be enrolled for this

study after giving informed consent. Criteria for exclusion included (a) ingestion of

antibiotics, bismuth, or proton-pump inhibitors within the prior 4 weeks (b) use of

non-steroidal anti-inflammatory drugs within the prior 4 weeks (c) patients with

allergic history to the medications used (d) patients with previous gastric surgery (e)

the coexistence of serious concomitant illness (for example, decompensated liver

cirrhosis, uremia), and (f) pregnant women. The H. pylori infection was defined as

positive results of both rapid urease test and histology.

**Selection of patients**

The *H. pylori*-infected outpatients who fail first-line eradication therapies will be randomly assigned to undergo the 10-day sequential (esomeprazole 40 mg bid., amoxicillin 1 g bid.for 5 days and followed by levofloxacin 500 mg qd and metronidazole mg tid for 5 days) or 10-day EAL groups (40 mg esomeprazole twice daily, 1 g of amoxicillin twice daily and 500 mg of levofloxacin once daily for 10 days respectively) Esomeprazole and amoxicillin are taken one hour before breakfast and dinner, metronidazole 500 mg after breakfast and dinner , levofloxacin once daily after breakfast. Patients are asked to return on the 2nd week to assess drug compliance and adverse events. Patients with peptic ulcers in initial endoscopy will receive an additional three weeks of esomeprazole 40 mg orally once daily, while patients with gastritis only take three weeks of antacid following eradication therapy. To assess eradication efficacy, repeat endoscopy with rapid urease test and histological examination will be performed at four to eight weeks after the end of anti-*H. pylori* therapy. For patients who refuse follow-up endoscopy, urea breast test is used to confirm *H. pylori* status. The absence of *H. pylori* after a previous eradication therapy is defined as (1) negative results of both rapid urease test and histology, or (2) a negative result of urea breath test at 4-8 weeks. The technicians who perform the *H. pylori* tests (rapid urease test and urea breath test) or fill in the questionnaires as well as the pathologists are blinded to the eradication regimens the patients received. All participants will give written informed consent.

**Questionnaire**

A complete medical history and demographic data are obtained from each patient, including age, sex, and medical history, history of smoking, alcohol, and coffee or tea consumption. Smoking is defined as consumption of one pack or more of cigarettes per week. Adverse events are prospectively evaluated such as abdominal pain, diarrhea, constipation, dizziness, taste perversion, headache, anorexia, nausea, vomiting and skin rash that bother patients’daily life quality. Compliance is checked by counting unused medication at the completion of treatment. Poor compliance is defined as taking less than 80% of the total medication (20).

**Objectives**

In the current study, we test the hypothesis that the 10-days sequential therapy can attain a better eradication rate than 10-day EAL as second-line therapy.

**Outcomes**

The primary endpoint of our study is successful eradication of *H. pylori.* There are additional analyses on adverse events during therapies.

**Diagnosis of Helicobacter pylori infection**

**Rapid urease test**

Rapid urease test: Gastric antrum biopsy specimens collected during endoscopy are tested using a commercial rapid urease test (Pronto Dry, Medical Instrument Corp, Solothurn, Switzerland) (21). The results of the rapid urease test are interpreted as positive if the color of the gel turned pink or red 1 h after examination at room temperature.

**Urea breath test**

The urea breath test is performed according to our previous studies [22]. The cut-off value is set at 4·8 ‰ of δ^13^CO_2_. Staffs are blinded to the *H. pylori* status performed the tests.

**Culture and antimicrobial resistance**

One antral gastric biopsy specimen is obtained for isolation of *H. pylori*, using previously described culture methods [23]. *H. pylori* culture is done by rubbing the specimens on the surface of a Campy-BAP agar plate [Brucella agar (Difco, Sparks, MD, USA) + IsoVitalex (Gibco, Grand Island, NY, USA) +10% whole sheep blood]. Then, they are incubated at 37°C under microaerobic conditions (5% O2, 10% CO2 and 85% N2) for 4–5 days. The results are considered positive if one or more colonies of gram-negative bacilli with positive oxidase, catalase and urease tests are found. The *H. pylori* strains are tested for tetracycline, metronidazole and clarithromycin susceptibility using the E-test (AB Biodisck, Solna, Sweden). *H. pylori* strains with a minimal inhibitory concentration (MIC) value > 4μg mL−1, 8μg mL−1 and > 1μg mL−1 are considered to be resistant to tetracycline, metronidazole, and clarithromycin, respectively.

**Randomization**

A ‘random sequence’ is set by computer-generated randomization list. The combining blocking and stratified randomization method is applied in each group. We set separate randomization within each of two subsets of participants. Every set block is composed of 6 participants. Our statistician will draw a computer generated randomization list and clinicians decided patients’ suitability to be enrolled in this study after careful rechecked process. The data collection and laboratory analyses are considered completed after the entry of the patient. Throughout the recruited process, all participants and doctors are blinded to treatment assignment for the duration of this study.

**Statistical analysis**

The primary outcome variables will be the rates of eradication, adverse events and compliance. Chi-square test with or without Yates correction for continuity and Fisher’s exact test are used where appropriate to compare the major outcomes between groups using the SPSS program (Statistical Package for the Social Sciences version 10-1, Chicago, IL, USA). Eradication rates are evaluated by Intention-to-treat (ITT) and per protocol (PP) analyses. ITT analysis includes all

randomly assigned patients who had taken at least one dose of study medication. Patients whose infection status is unknown following treatment were considered treatment failures for the purposes of ITT analysis. The PP analysis excludes patients with unknown H. pylori status following therapy and those with major protocol violations. A P-value less than 0.5 is considered statistically significant. To determine the independent factors affecting the treatment response, 12 clinical and bacterial parameters will be analysed by univariate analysis.

**Study Protocol flow chart**

10-day sequential

esomeprazole 40 mg bid., amoxicillin 1 g bid.for 5 days and followed by levofloxacin 500 mg qd and metronidazole 500 mg tid for 5 days

Patients who fail 1^st^ line

*H pylori* eradication using the standard triple therapy

esomeprazole 40 mg bid., amoxicillin 1 g bid., and levofloxacin 500 mg qd for 10 days

10-day EAL groups sequential

Day 0

Day 10

4-8 wk

**Questionnaire**

E

**Endoscopy**

**Gastric biopsy for histology and *H, pylori***

**examination**

**and culture**

**Rapid urease test**
